# A cost-effectiveness analysis of COVID-19 critical care interventions in Addis Ababa, Ethiopia: a modeling study

**DOI:** 10.1186/s12962-023-00446-8

**Published:** 2023-06-26

**Authors:** Senait Alemayehu Beshah, Arega Zeru, Wogayehu Tadele, Atkure Defar, Theodros Getachew, Lelisa Fekadu Assebe

**Affiliations:** 1 Health System and Reproductive Health Research Directorate, Ethiopian Public Health Institute, Addis Ababa, Ethiopia; 2 Department of Global Health and Population, Harvard T.H. Chan School of Public Health, Harvard University, Boston, MA USA

**Keywords:** COVID-19, Cost, Cost-effectiveness, Critical. Invasive, Noninvasive

## Abstract

**Objective:**

To estimate and compare the cost-effectiveness of COVID-19 critical care intervention approaches: noninvasive (oxygen without intubation) and invasive (intubation) management in Ethiopia.

**Methods:**

A Markov model is used to compare the costs and outcomes for non-invasive and invasive COVID-19 clinical interventions using both primary and secondary data sources. Healthcare provider costs (recurrent and capital cost) and patient-side costs (direct and indirect) were estimated and reported in United States Dollars (US$), 2021. The outcome measure used in this analysis was DALYs averted. Both the average cost-effectiveness ratio (ACER) and incremental cost-effectiveness ratio (ICER) were reported. One-way and probabilistic sensitivity analyses were applied to assess the robustness of the findings. The analysis is conducted using Tree Age pro health care software 2022.

**Result:**

The average cost per patient per episode for mild/moderate, severe, noninvasive, and invasive critical management was $951, $3449, $5514, and $6500, respectively. According to the average cost-effective ratio (ACER), non-invasive management resulted in $1991 per DALY averted, while invasive management resulted in $3998 per DALY averted. Similarly, the incremental cost-effective ratio (ICER) of invasive compared to noninvasive management was $ 4948 per DALY averted.

**Conclusion:**

Clinical management of critical COVID-19 cases in Ethiopia is associated with a significant financial burden. Invasive intervention is unlikely to be a cost-effective COVID-19 intervention in Ethiopia compared to noninvasive critical case management using a willingness to pay threshold of three times GDP per capita.

**Supplementary Information:**

The online version contains supplementary material available at 10.1186/s12962-023-00446-8.

## Introduction

Globally, the COVID-19 pandemic affected 235 countries/territories causing 78,422,954 cases and 1741,204 deaths (CFR = 2.22%), between December 2019 and December 2020. The United States of America (USA) reported the highest number of cases [1].

In Africa, 57 countries/territories have reported COVID-19 cases and the pandemics as December 2020, a total of 2649440 cases and 61971 deaths were reported across the continent (CFR = 2.34%). South Africa reported the highest number of cases (1473700) with a CFR of 2.68% followed by Morocco (474966 cases) with a CFR of 1.67%.

Ethiopia reported the highest number of COVID-19 confirmed cases in East Africa and ranked third next to South Africa and Morocco. Ethiopia confirmed its first case of COVID-19 on 13 March 2020, two days after the WHO declared a pandemic of the disease. As of 01 April 2021 COVID-19 pandemic preparedness and response daily situation report for Ethiopia, the country tested 2365187 suspects, of whom 208961 (9%) cases had been confirmed positive and of these 2890 (CFR = 1.4%) died and 159436 (31.1%) recovered and more than 90% cases were managed in Addis Ababa [2].

A study conducted in Japan showed that COVID-19 hospitalized patients’ mortality rates vary across severity status and range from 1.4% to 19% (mild to severe cases) and as high as 62% among those requiring critical care, and those who are older and with comorbidities have greater fatality rates [3].

The COVID-19 severity classification includes (1) Critical COVID-19 is defined as for acute respiratory distress syndrome (ARDS), sepsis, septic shock, or other conditions that would normally require the provision of life-sustaining therapies such as mechanical ventilation (2) Severe COVID-19 is defined as oxygen saturation < 90%, on room air, signs of severe respiratory distress (3) Non severe COVID-19 is defined as the absence of any critical or severe COVID-19,and it includes asymptomatic, mild, and moderate case [4] Managing the mild to moderate COVID-19 cases was maintaining standard infection prevention and control procedures, using empirical oral antibiotics without oxygen support.

When a patient is in critical condition, noninvasive interventions such as continuous positive airway pressure (CPAP), high flow nasal oxygen (HFNO), was given and if such measures are unsuccessful, intubation and mechanical ventilation should be considered [5]. The widespread corona-virus (COVID-19) pandemic presents unanticipated challenges to healthcare systems around the world. Among these challenges is a shortage of life-saving supplies including diagnostic tests, ventilators, and personal protective equipment (PPE) [6].

Moreover, patients with critical COVID-19 often require costly treatment such as mechanical ventilation and extracorporeal membrane oxygenation, potentially substantially increasing healthcare costs. In Iran, the average medical cost for COVID-19 treatment was estimated at USD 3,755 per critical case. This study asserted that the high incidence of COVID-19 poses a substantial economic burden to the health system [7]. In Africa, there are very few countries that have assessed COVID-19 treatment cost, and the results from these assessments vary among countries [8]. For example, the estimated unit cost per day for COVID-19 treatment in Kenya in 2020 at home based isolation care, mild /moderate, severe and critical care were USD 18.89, USD 63.68, USD 124.53, and USD 599.51, respectively [9]. Another study conducted in South Africa showed COVID-19 management in an intensive care unit cost USD 844.88 per day at the government hospital [10].

A study conducted in Ethiopian revealed that the cost of COVID-19 treatment varied by disease severity: the mean cost per episode for the moderate, severe, and critical cases were USD 1266 (998–1534), USD 1545 (1413–1677), and USD 2637 (1788–3486), respectively [11]. Ethiopia is a low-income country with a Gross Domestic Product (GDP) per capita of USD953 in 2019 [12] and a per capita health expenditure of about USD33 in 2016/17 [18]. The COVID-19 inflicted an enormous burden in terms of the cost of inpatient, outpatient, and other health system costs, as well as productivity losses [13].

COVID-19 has a higher impact on the health system and supply chain stock personal income level, and health system. A significant economic impact has already occurred across the world including in Ethiopia, due to the condensed, loss of life and work, business closures, trade disruption, and decimation of the tourism industry [14]. The study conducted in Wuhan suggests that during COVID-19 critical management avoid invasive (IV) and utilize noninvasive (NIV) at the early stage of respiratory failure until invasive is inevitable [15], Study also presented that noninvasive ventilation was successful in patients with moderate to severe ARDS [18, 19].

In mechanically ventilated patients, mortality of COVID-19 cases has ranged from 50 to 97% [17]. In New York, a cohort study suggests that the death rate in invasive mechanical ventilation for COVID-19 interventions is as high as 88.1%. Based on these high mortality rates, there has been speculation that this disease that ARDS mechanical ventilation approach may not be as effective in reducing lung damage [18]. More studies are required worldwide to identify that invasive intervention outcome because it may not reduce the mortality in COVID-19 patients [15]. Thus, this study aimed to evaluate the cost-effectiveness of COVID-19 critical care invasive and non-invasive management in Addis Ababa, Ethiopia. The study provides recommendations to help clinicians and policymakers allocate resources more efficiently during the management of COVID-19 critical cases.

## Methods

This study was conducted in Addis Ababa, Ethiopia. Addis Ababa is the capital city of Ethiopia, and has an area of 540 square kilometers, managing a high load of COVID-19 cases during the study period [19]. Based on the caseload, we collected primary data from Eka Kotebe hospital, the first COVID-19 case treatment site in Ethiopia and 4 health centers (Caffe, Addis ketma, Kolefe and Kirqose COVID 19 treatment center). A sample of 210 COVID-19 admitted patients (age 18 and over) was randomly selected. A cross-sectional study design was employed for primary data collection. The top-down and bottom-up micro-costing ingredient-based approach was used to estimate the average cost per episode of managing severe, and critically ill COVID-19 cases. This costing approach considers global spending at a central level to allocate costs to each intervention [2].

### Study design

This study was a full economic evaluation using the Markov model to provide relevant cost and effectiveness information on COVID-19 critical case management in Ethiopia.

### Comparators groups

The study compared non-invasive and invasive COVID-19 critical case management. Non-invasive ventilation (NIV) refers to the administration of ventilator support through a face mask, nasal mask, or helmet without using an invasive artificial airway (endotracheal tube or tracheostomy) [20]. During critical care management, if the patient did not respond within a non-invasive ventilator it may move to invasive mechanical ventilation, which is positive pressure delivered to the patient's lungs via an endotracheal tube or a tracheostomy tube [17, 21]. The cycle length used in this model was monthly, and the costs were per episode. A half-cycle correction is applied to assume that the transition to events occur halfway through a cycle.

### Time horizon

The study was conducted with a lifetime horizon. According to the world life expectancy, the Ethiopian life expectancy was 65 years. Since this study started with ages above 18 years, we run the model in 47 cycles to find the difference in the life expectancy [22].

### States, transitions and disease progression

**Fig. 1:**
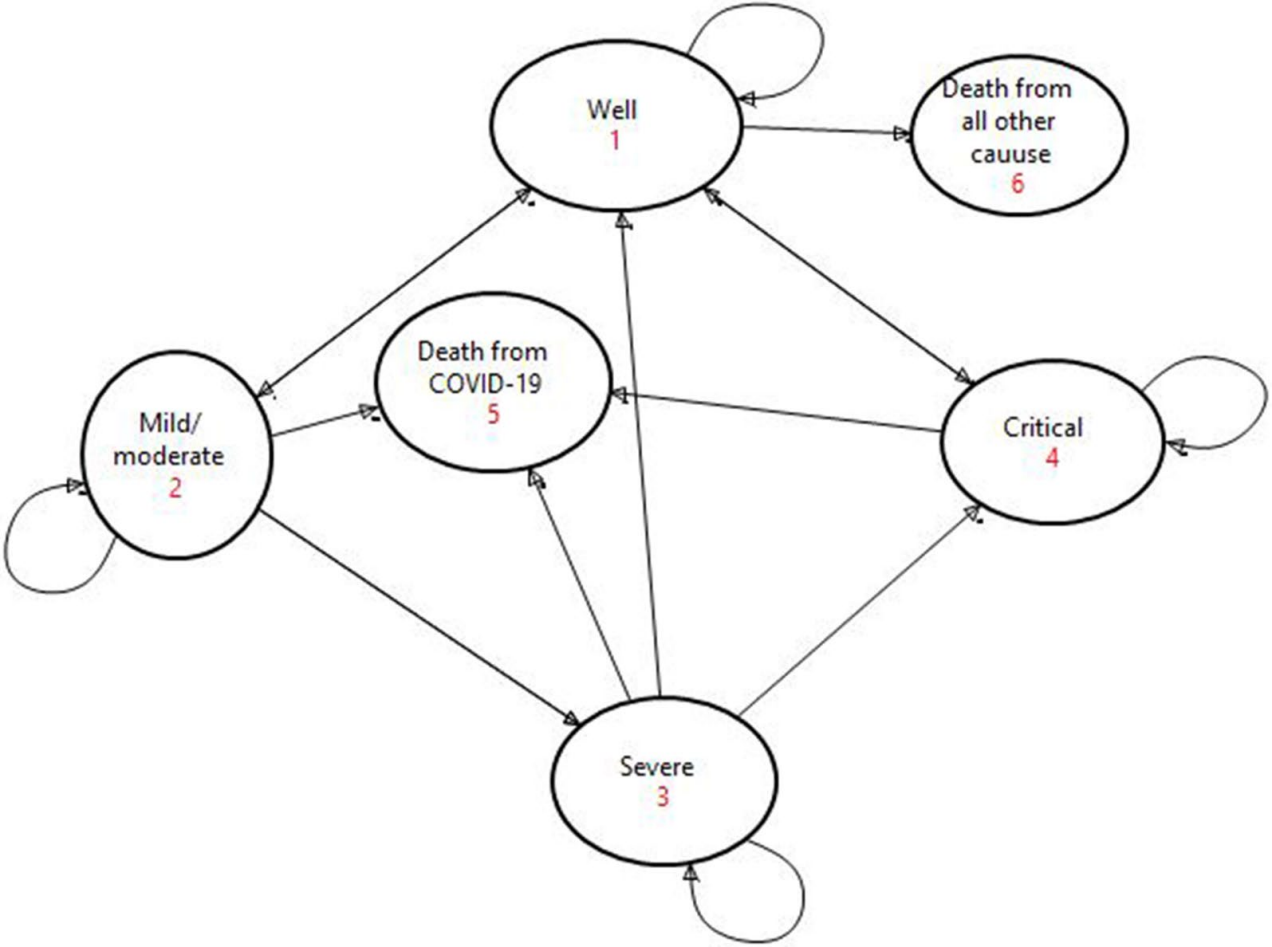
Markov tree state-transitions diagram

Initially, all individuals would be ‘well’ or susceptible to COVID-19. A person from a ‘well’ state would be infected with a certain probability of COVID-19 about 80%, 15%, and 5% mild and moderate, severe, and critical cases respectively [23]. And patients with comorbidities had a higher death rate among COVID-19 hospitalized patients. If the patient was in critical condition, the likelihood of death was 87.2%. Besides, 80% of mild /moderate COVID-19 cases recovered from the disease [24]. Based on this fact, the Markov model was structured in six health states: Well, mild /moderate, severe, critical, death from COVID-19, and death from other causes. The health states and transition states in any given time interval, consider the individual in only one health state with mutually exclusive states (Fig. 1). The transition probabilities are the probabilities in which a subject is moving from one state to another state within a given cycle length [25] and it was determined by published data [26].

### Target Population

The sample source of this study was a cohort of patients enrolled at the COVID-19 treatment center and home-based isolation care who were registered from January 1-May 31, 2021 in Addis Ababa.

### Cost and effectiveness data sources

The data collection instrument is adapted from the, WHO, 2017 methodology, costing investigation [27]. The costing was conducted based on the healthcare and patient perspectives. To identify the types of resource inputs, we reviewed the Ethiopia COVID-19 clinical management guidelines, published articles and expert opinions; besides we also used the WHO COVID-19 Essential Supplies Forecasting tool [28]. Besides, the direct medical and nonmedical costs, we also included indirect costs (i.e., productivity loss due to COVID-19). We used primary and secondary data sources for the cost-effectiveness analysis model (Table 1).

**Table 1:** Probability and cost (USD 2021) used for the cost- effectiveness analyses model

Probability data	Base value	Range	Dis^a^	Data source
Well person to Critical	0.05	0.04–0.06	Beta	[4]
Well person to Severe	0.15	0.12–0.18	Beta	[4]
Well person to MIMO	0.8	0.64–0.96	Beta	[4]
Mortality from invasive case	0.30	0.5–0.8	Beta	[18]
Disability weight of COVID-19 in mild/moderate	0.051	0.032–0.074	Beta	[27, 27]
Disability weight of COVID-19 in severe case	0.13	0.09–0.19	Beta	[19, 26]
Disability weight of COVID-19 in critical case	0.41	0.27–0.56	Beta	[19, 26]
COVID -19 death from MIMO	0.03	0.024–0.036	Beta	Primary
COVID-19 death from Severe	0.12	0.096–0.14	Beta	Primary
COVID -19 death from invasive	0.97	0.89–0.99	Beta	Primary [17, 18]
COVID -19 death from none-invasive	0.33	0.264–0.396	Beta	Primary^[15]^
COVID -19 recovery from severe	0.88	0.70–1	Beta	Primary
COVID -19 recovery from MIMO	0.97	0.776–1	Beta	Primary
COVID -19 recovery from invasive	0.02	0.016–0.024	Beta	Primary
COVID- 19 recovery from noninvasive	0.67	0.53–0.80	Beta	Primary
COVID- 19 cost for severe	3449	2759–4139	Gama	Primary
COVID -19 cost for noninvasive	5514	4411–6616	Gama	Primary
COVID -19 cost for invasive	6500	5200–7800	Gama	Primary
Discounting for costs and effectiveness	0.03	0.01–0.06	Beta	[28]
COVID 19 cost for mild/moderate	951	761–1142	Gama	Primary

^*a*^*Dis distribution, MIM mild/moderate*

### Cost and effectiveness measures (CEA)

The cost analysis was completed on Excel 2021 spreadsheet [29] and SPSS version 25. Productivity loss or the indirect cost of the patients is the gross wage is the unit of value before COVID-19 illness, income and was assessed based on the human capital approach (HCA) [36].

The patient direct nonmedical costs were related to patient costs for COVID-19 services and costs incurred to access these services. These are costs related to round-trip transportation, nutritional foods, and lodgings costs which are quantified from the patient face-to- face interview. The number of visits and the COVID-19 hospital-admitted patient's food costs were estimated based on the last study done on Multidrug- resistant tuberculosis (MDR TB) admitted food costs because the COVID-19 patients' food also prepared by the government with nutritious content [25].

To calculate the personnel cost, the staff-to-patient ratio was used based on the opinions of government experts working at appropriate staffing levels of care for COVID-19 management [4]. The number of hours worked per day for all staff in the facility including support staff (laundry, spryer, administration) was multiplied by the probability that the staff would provide care to patients (i.e., staff time per patient per day). The personnel who do not have a staff-to-patient ratio were estimated based on the exact hours used per case. Finally, staff time per patient per day was multiplied by the daily staff salary, allowances, and duty time payments rate to estimate the staff daily cost per patient, then multiplied by per episode based on the severity of the cases.

All services were provided free of charge thus, costs for drugs, investigation and medical supply, and personal protective equipment costs were collected from other government hospitals and all costs were assessed based on the average unit cost per patient per day used/consumed to sum up the cost per episode and multiplied by the number of tests/drugs supply per day per severity. Oxygen cost was based on the expert opinion of the patient on moderate flow oxygen therapy is 6 to 15 L per minute using a simple face mask, reservoir mask and venture mask average consumption of a single patient is 40 L 2 and a half cylinders per day per patient. High-flow oxygen therapy is 20 to 60 L per minute using mechanical ventilators and the average consumption of a single patient is 40 L 6 cylinders per day per patient. Noninvasive mode pressure supports ventilation average consumption is 40 L 12 cylinders per day per patient.

To estimate the cost of buildings, we measured the total building area in the facility and multiplied it by the local market rental rate estimate, which was based on the average rental price of several buildings in the neighborhood. The cost of vehicles was estimated using their rental equivalents. We calculated the equivalent annual cost (and adjusted for the period) of equipment using the initial capital outlay over the lifetime of the asset. Equipment costs were retrieved from the Ethiopian Pharmaceutical Supply Agency and facility finance department and World Health Organization (WHO) COVID-19 essential supplies forecasting tool and then all capital resources such as the furniture, mechanical ventilator ICU beds, and other medical equipment costs were annuitized based on the useful life-years, and all cost were discounted at 3% rate initial unit price, besides that, we consider the consumer price index for annual inflation rate [31].

All the unit cost per day per patient was multiplied by the average stay per severity to generate the cost per episode per patient for COVID-19 case management based on the facility. Valuation involves two steps of costing: measuring the quantities of resources utilized with their unit prices followed by valuing the resources using Ethiopian Birr and converting it to USD, with the exchange rate of 41.5 Birr during the study period. The prices were adjusted for inflation using a consumer price index of the year 2021 as a base year cost, and we reported all costs in 2021 US Dollars. The average length of stay in the health centers was 14, and 16 days for mild /moderate and severe cases, however, this number of days could be a bit longer within the hospitals i.e., 18, 19, and 21 for mild/moderate, severe, critical case patients. Finally, unit cost was estimated based on the disease’s severity and multiplied by the average length of stay per the facility^10^.

### Building a model for effectiveness measures

Markov state transition model using Tree Age pro health care software 2022 was used for the cost-effectiveness analyses [41]. The health outcome measure is Disability-Adjusted Life Years (DALYs), and calculated by adding YLL and YLD of COVID-19 treatment [11]. The Disability- weight for COVID-19, and years of life lost due to premature mortality, are sourced from: the global burden of disease study and the WHO life table [27]. The incremental cost-effectiveness ratios (ICER) of COVID-19 treatment in Ethiopia are being evaluated, and the resulting ICER is characterized using Willingness-To-Pay (WTP) thresholds.

The ICER were reported comparing the strategy with a ‘do-nothing’ choice besides the average cost-effectiveness ratio was reported in cost per DALYs averted [30, 32]. This approach uses a pre-defined definition of value, the WTP threshold, to guide decision-making [33, 35]. This study analysis was evaluated based on an intervention that provides value relative to an existing intervention based on the COVID-19 critical (invasive and noninvasive) death and recovery per episode (with value defined as cost relative to health outcome) DALYs [34, 36]. Furthermore, probabilistic sensitivity analysis was used to analyze the cost-effectiveness using scatterplot and acceptability curve to test the stability of the model results.

### Sensitivity analysis

To evaluate the robustness of the results both one-way (tornado analysis) and probabilistic sensitivity analysis (PSA) were conducted. A sensitivity analysis employed in this study would help to determine how different values of an independent variable impact cost-effectiveness analysis under a given set of assumptions.

### Ethical approval and consent to participation

This study was approved by the Institutional Review Board (IRB) of Ethiopia public health institutes (EPHI-IRB-360-2021) on July 23/2021 and all data were collected with informed consent.

### Patient and public involvement

This study did not involve patients or the public in the design, implementation, or dissemination.

## Result

### Socio-demographic characteristics

Among the 210 participants in the study, the majority was male (65.2%). From the respondents 66 (31.4%) of the participants were between 18 and 35 years old, 96 (45.7%) were between 36 and 55 years old, and 22.8% were more than 55 years old. The findings also revealed that 123 (58.6%) of the participants were married, 40 (19%) were single, and the remaining 21 (10.1%) were divorced or widowed (Additional file 1: Table S1).

### Estimation of COVID-19 intervention cost

The direct non-medical and indirect (productivity loss) cost for COVID-19 intervention ranged from USD 16.8 to USD 82.52 per day for patient in HBIC and ranges from USD 235.91 to USD 1733.13 for patient in hospital critical care intervention (Additional file 1: Table S2).

Based on the health care perspective the average cost of the health system per episode based on the ingredients approach was USD34.13 to USD 4,767.54for home-based isolation care and critical noninvasive patient, respectively. The average patient daily cost was USD 2 for HBIC to USD 277 for invasive management. The supply cost was the major cost driver, ranging from USD 2.4 for HBIC to USD 151 for critical invasive management and followed by the personal cost of USD 4 for mild/moderate to USD 51 for critical case per patient per day. However, the building cost was the least cost when compared to other cost in this study (Additional file 1: Table S3).

During the COVID-19 case management the leading costs were all supply cost 64% (oxygen, drugs, PPE and lab investigation) costs and seconded by the personnel cost 22% and 9% for costs for equipment’s the least cost was 5% were invested for building as (Additional file 1: Figure S1).

Furthermore, the societal (patient and health system) perspective average cost per episode when we manage COVID-19 in the hospital was USD 951.83, USD 3449.9, USD 5514, and USD 6500.67 for Mild /moderate, severe, critical care invasive and noninvasive management per episode, respectively, Table 2.

**Table 2:** Cost per episode for COVID-19 treatment, based on severity of cases (Patient and health system) perspective (2021 USD)

Health facility	Severity	Cost per day	Cost per patient per episode			
		Mean	Mean	SD	Min.	Max.
Health Center	Mild/moderate	38	546	109	437	655
	Severe	93	1442	288	1154	1731
Hospital	Mild moderate	56	951	190	761	1142
	Severe	184	3449	689	2759	4139
	Critical noninvasive	270	5514	1102	4411	6616
	Critical invasive	309	6500	1300	5200	7800

*Cost was in 2021 USD*

Min minimum, *Max* maximum

### Probabilistic sensitivity analysis (PSA)

The average cost-effective ratio (ACER) implies that the noninvasive mechanical ventilator intervention would need USD 1991 per DALYs averted. The invasive mechanical ventilator intervention would require USD 3998 per DALYs averted. The invasive strategy has ICER of USD 4948 per DALYs averted compared to the noninvasive as shown in Table 3, which is no cost-effective strategy, as the ICER is greater than three times GDP per capita per DALYs averted in Ethiopia.

**Table 3:** Cost-effectiveness Ratio per DALYs averted all referencing common baseline

Cost, effectiveness, average and incremental cost-effectiveness ratio per DALYs averted						
Strategy	Cost (USD)	Incremental cost	Eff (DALYs)	Incremental Eff	ACER	ICER
Noninvasive	6990.143		3.511		1991	
Invasive	10,887.619	3897.476	2.723	0.788	3998	4948

*USD* United States dollar, *DALYs* Disability adjusted life years, *Eff* Effectiveness

**Fig. 2:**
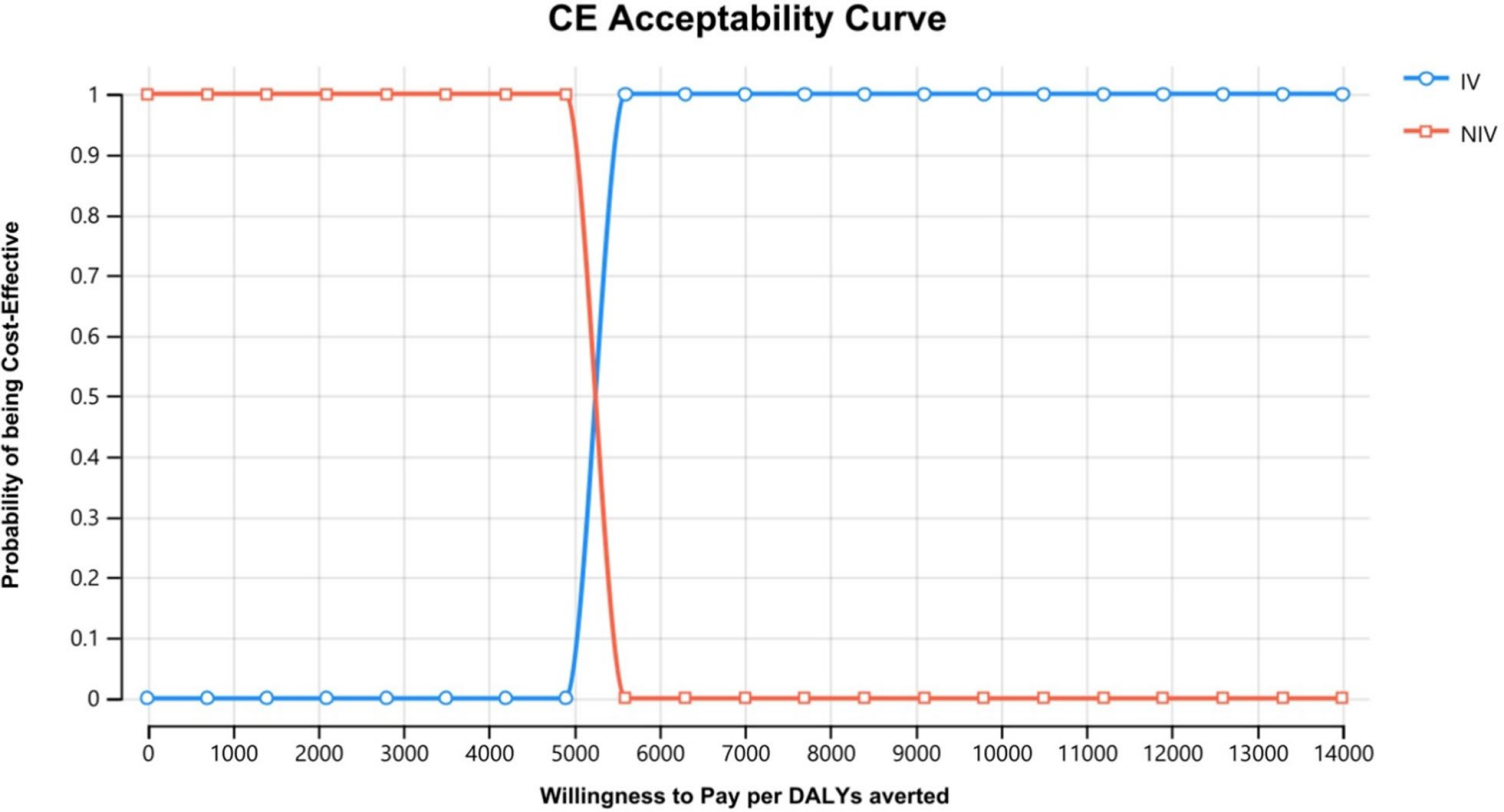
Cost-effectiveness acceptability curve

Figure 2 shows the graph plots of a range of cost-effectiveness thresholds (Willingness to Pay per DALYs averted) and the cost-effectiveness acceptability curve. If a decision-maker looking at these data will have a maximum willingness to pay of $ 5500 there is a 50–50 chance that the two option was cost-effective (50% acceptability of both interventions at this threshold), however, for invasive intervention, it is not a cost-effective option at a willingness to pay threshold compare with less than three times GDP per capita per DALYs averted in Ethiopia. The incremental CER was most influenced by the probability of recovery from mild to moderate cases that leads to ICER per DALY averted from 5000 to 7000 followed the probability of recovery from invasive mechanical ventilator in ICU that leads to ICER per DALY averted from 5000 to 6000.

**Fig. 3:**
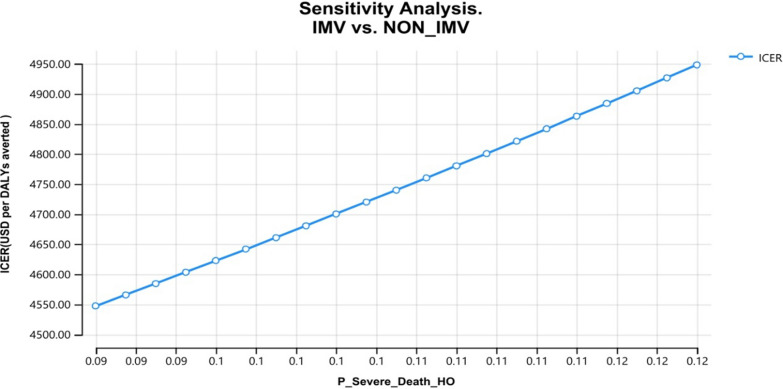
Probability of COVID 19 death from severe cases at hospital

Use of the selected one-way sensitivity analysis lists of variables is essential to illustrate the impact of the sensitivity analysis. According to the findings, among the COVID-19 patients who required extensive hospitalization, about 12% of cases were recovered using the $4950 invested presented as Fig. 3.

**Fig. 4:**
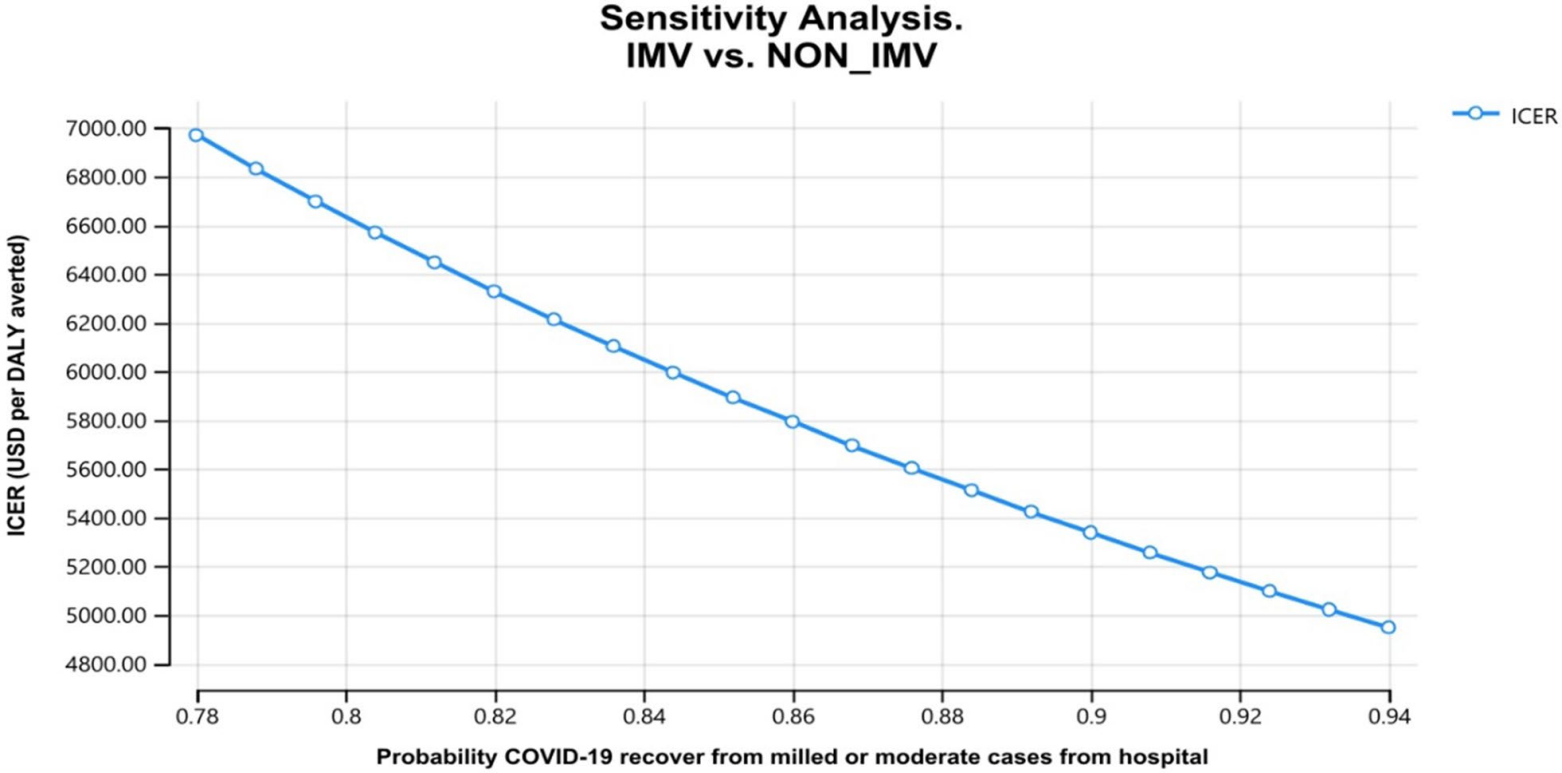
Probability of mild /moderate case recover at Hospital

The results in Fig. 4 show that 94% of COVID- 19 recovered from mild/moderate cases, which were managed at the hospital level. As a result, the cost-effectiveness ratio decreases as the probability of transition to recovery increases.

## Discussion

This study aimed to compare the cost and cost-effectiveness of COVID-19 critical care case management in invasive (IV) or intubated and noninvasive if not intubated (NIV) using a full economic evaluation. This study is the first of its kind in Ethiopia, since there is no published cost-effectiveness study. The study showed that the invasive intervention is unlikely to be a cost-effective intervention in Ethiopia when comparing noninvasive COVID-19 critical case management with a willingness to pay a threshold of three times GDP per capita.

Therefore, this study can inform policy decisions that have to be made on how best to allocate scarce public resources to maximize population health during the pandemic. For example, should resources be spent scaling up the provision of high-level non-invasive and/or invasive oxygen therapies? Lengthwise, further budget impact analysis and affordability studies will be needed. This study also notifies for physicians to reconsider intubation in COVID-19 critical care management because during intubation with a high cost fewer DALYs averted. The last study also confirmed that the physician will be focused on the intervention of noninvasively ventilated patients with COVID- 19 ARDS, as, showed that NIV was effective in almost half of the patients. And wider use of NIV potentially helps reduce progressive and probably avoid intubation in numerousus patients [37].

In this study in resource scarcity, Ethiopia will achieve better saving resources and better health outcomes during COVID-19 critical case management if utilize non-invasive intervention than invasive. There was agreement among studies that the NIV had an obvious beneficial effect on ARF among COVID-19 patients [38].

In this finding the cost was raising based on the severity from mild /moderate to the critical invasive case, this may be if the patient was more severe and consume more oxygen and more drugs based on the length of stay. These results were comparable to the last study. The highest average daily cost was estimated for ICU admissions and the lowest, for general ward-based care [39].

In this study the non-invasive intervention was cost-effective based on the Ethiopia's three times GDP per capita, likewise, the last study also suggest the non-invasive intervention clinically useful until future research provided [16].

During COVID-19 ICU management, the invasive intervention was comparatively more expensive because it consumes high cost (which is the function of the unit cost of the invasive mechanical ventilator, high qualified personnel cost, PPE, oxygen including all supply costs, and length of stay). The associated study showed that the critical care of COVID-19 patients’ admission to ICU was poor (unlikely to be a lifesaving intervention) outcome and consume high cost [10].

In this study the invasive intervention was low DALYs averted with the high-cost theses may be prolonged time intubation lead to lung infection, this result was similar with the other study. During COVID -19 critical care invasive (intubated) intervention was high mortality when compared to the noninvasive this may there remain a valid concern that the use of NIRS may prolong the time to intubation and lung protective ventilation in patients with more advanced disease, thereby worsening respiratory mechanics via self-inflicted lung injury [16] and other previous study showed also during COVID-19 critical intervention the mechanically ventilated patients the progress was poor, or mortality was high [40]. Another study also showed that the critical care management of COVID-19 consumed high cost and low DALYs averted [41]. This study is the primary of its kind in Africa that compared the cost and cost-effectiveness of COVID-19 critical case intervention.

The unavailability of cost data, and tried to alleviate this challenge using scientific references to make the study as a springboard for future studies in economic analysis of COVID-19 and fulfills data requirement/gap for a low-income country like Ethiopia particularly for costs arising time lost in seeking care. Furthermore, the following can be considered as strengths.

- This study is the first in Africa that compare the cost and cost-effectiveness of COVID-19 critical case interventions.
- Costs were thoroughly evaluated from a societal perspective, including both patient and provider perspectives.
- Analysed the primary cost data for COVID-19 treatment from HBIC, health centre and hospital and filling the data gap for provider and patient costs in COVID-19 management in Ethiopia.
- The study’s findings provide valuable information for mobilizing Ethiopia's capital and consumable resources for COVID-19 budgeting and planning, as well as offering evidence-based guidance for physicians involved in critical interventions for COVID-19.

Some of the limitations are:

- There were no published studies to compare our cost and cost-effectiveness result.
- The exclusion of COVID-19 contacts tracing cost data (before-after COVID-19) may underestimate the unit cost of treatment.
- This study's limited focus on a few numbers of healthcare facilities may limit its representativeness to the larger context of COVID-19 treatment in Ethiopia.

## Conclusion

These results indicated that the critical case of the disease extremely carries a high cost and low effect or outcome in this event. It is projected that the high prevalence rate of COVID-19 has been imposing a heavy economic burden on the health system and patients and even in the country. The critical invasive mechanical ventilator was unlikely to be cost-effective within three times the GDP per capita of Ethiopia (willingness to pay threshold). Thus, high awareness needs to prevent to minimize the amount of cost because the worsening COVID-19 burden could add to the rising loss of life, resources, and productivity.

## Supplementary Information

**Additional file 1: Figure S1.** Ingredients based dally costs of COVID -19 management. **Table S1.** Study participants demographic characteristics. **Table S2.** Estimation of COVID-19 treatment cost by the level of severity and treatment setting per patient inpatient perspective. **Table S3.** Cost for COVID-19 treatment by ingredient, level of severity and treatment setting per patient in health care perspective.

## Data Availability

All data supporting results will be available on official request to the corresponding author.

## Abbreviations

CFD: Case fatality rate
ECG: Electrocardiography
CPAP: Continuous positive airway pressure
EPSA: Ethiopia pharmaceuticals supply agency
ETB: Ethiopian birr
HDU: High dependency unit
PPE: Protective equipment
HBIC: Home based isolation care personal
NG: Nasogastric
ICU: Intensive care unit
IV: Intravenous
USD: Untied Studs Dollar
