# Supplementary material for: A cost-effectiveness analysis of COVID-19 critical care interventions in Addis Ababa, Ethiopia: a modeling study
Source: Cost Eff Resour Alloc. 2023 Jun 26;21:40. doi: 10.1186/s12962-023-00446-8 (PMC10291773; doi:10.1186/s12962-023-00446-8)
Supplement: Supplementary file 1 — Additional file 1: Figure S1. Ingredients based dally costs of COVID -19 management. Table S1. Study participants demographic characteristics. Table S2. Estimation of COVID-19 treatment cost by the level of severity and treatment setting per patient inpatient perspective. Table S3. Cost for COVID-19 treatment by ingredient, level of severity and treatment setting per patient in health care perspective. [file 12962_2023_446_MOESM1_ESM.zip › Supplementary Tables and figure/Supplementary Tables and figure/Figure S1.docx]

**Figure S1. Ingredients based dally costs of COVID -19 management (US$2021).**
