# Supplementary material for: A cost-effectiveness analysis of COVID-19 critical care interventions in Addis Ababa, Ethiopia: a modeling study
Source: Cost Eff Resour Alloc. 2023 Jun 26;21:40. doi: 10.1186/s12962-023-00446-8 (PMC10291773; doi:10.1186/s12962-023-00446-8)
Supplement: Supplementary file 1 — Additional file 1: Figure S1. Ingredients based dally costs of COVID -19 management. Table S1. Study participants demographic characteristics. Table S2. Estimation of COVID-19 treatment cost by the level of severity and treatment setting per patient inpatient perspective. Table S3. Cost for COVID-19 treatment by ingredient, level of severity and treatment setting per patient in health care perspective. [file 12962_2023_446_MOESM1_ESM.zip › Supplementary Tables and figure/Supplementary Tables and figure/Supplementary Table1.docx]

Table S1 Study participants demographic characteristics

| **Variable**  **Description** | | **Total** | |  |
| --- | --- | --- | --- | --- |
|  |  | **N** | **%** |  |
| Sex | Female | 73 | 34.8 | |
|  | Male | 137 | 65.2 | |
| Age | 18-25 | 20 | 9.5 | |
|  | 26-35 | 46 | 21.9 | |
|  | 36-45 | 57 | 27.1 | |
|  | 46-55 | 39 | 18.6 | |
|  | 56-65 | 37 | 17.6 | |
|  | 66+ | 11 | 5.2 | |
| Marital Status | Divorces | 9 | 4.3 | |
|  | Married | 123 | 58.6 | |
|  | Single | 40 | 19.0 | |
|  | Widowed | 12 | 5.7 | |
| Education Level | Post grad. | 17 | 8.1 | |
|  | Bachelor | 18 | 8.6 | |
|  | Diploma | 18 | 8.6 | |
|  | Certificate up to Level II | 48 | 20.0 | |
|  | Read and write | 40 | 19.0 | |
|  | Not attended Sch. | 11 | 5.2 | |
