# Supplementary material for: A cost-effectiveness analysis of COVID-19 critical care interventions in Addis Ababa, Ethiopia: a modeling study
Source: Cost Eff Resour Alloc. 2023 Jun 26;21:40. doi: 10.1186/s12962-023-00446-8 (PMC10291773; doi:10.1186/s12962-023-00446-8)
Supplement: Supplementary file 1 — Additional file 1: Figure S1. Ingredients based dally costs of COVID -19 management. Table S1. Study participants demographic characteristics. Table S2. Estimation of COVID-19 treatment cost by the level of severity and treatment setting per patient inpatient perspective. Table S3. Cost for COVID-19 treatment by ingredient, level of severity and treatment setting per patient in health care perspective. [file 12962_2023_446_MOESM1_ESM.zip › Supplementary Tables and figure/Supplementary Tables and figure/Supplementary Table2.docx]

Table S2 Estimation of COVID-19 treatment cost by the level of severity and treatment setting per patient in (2021 USD) patient perspective

| Patient Cost | HBIC | Health Center | | Hospital | | |
| --- | --- | --- | --- | --- | --- | --- |
|  |  | **Mild /mod** | **Severe** | **Mild Moderate** | **Severe** | **Critical** |
| Patient (Non-medical- Direct) Cost USD | 1.61 | 15.96 | 21.6 | 26.5 | 26.5 | 26.5 |
| Indirect (productivity loss) cost USD | 15.24 | 8.26 | 8.19 | 30.43 | 56.83 | 56.03 |
| Cost per Patient /day in USD | 16.85 | 24.14 | 29.79 | 56.93 | 83.33 | 82.53 |
| Cost per Patient/ episode USD | 235.914 | 338.24 | 475.2 | 1024.74 | 1583.27 | 1733.13 |

ETB: Ethiopian birr, USD: united states dollar
