# Supplementary material for: A cost-effectiveness analysis of COVID-19 critical care interventions in Addis Ababa, Ethiopia: a modeling study
Source: Cost Eff Resour Alloc. 2023 Jun 26;21:40. doi: 10.1186/s12962-023-00446-8 (PMC10291773; doi:10.1186/s12962-023-00446-8)
Supplement: Supplementary file 1 — Additional file 1: Figure S1. Ingredients based dally costs of COVID -19 management. Table S1. Study participants demographic characteristics. Table S2. Estimation of COVID-19 treatment cost by the level of severity and treatment setting per patient inpatient perspective. Table S3. Cost for COVID-19 treatment by ingredient, level of severity and treatment setting per patient in health care perspective. [file 12962_2023_446_MOESM1_ESM.zip › Supplementary Tables and figure/Supplementary Tables and figure/Supplementary Table3.docx]

Table S 3 Cost for COVID-19 treatment by ingredient, level of severity and treatment setting per patient in (2021 USD) health care perspective

| Ingredient’s cost | HBIC | Health Center | | Hospital | | | |
| --- | --- | --- | --- | --- | --- | --- | --- |
|  |  | **Mild /Moderate** | **Severe** | **Mild Moderate** | **Severe** | **Critical**  **NIV** | **Critical IV** |
| All supply cost | 2.4 | 12.04 | 47.89 | 14.3 | 86 | 111.42 | 151.3 |
| Personals |  | 4.18 | 10.6 | 5.4 | 24.7 | 51.10 | 51.1 |
| Equipment |  | 3.80 | 7.49 | 4.38 | 12.3 | 16.51 | 16.51 |
| Building |  | 2.15 | 3.06 | 2.319 | 4.527 | 8.147 | 8.14 |
| Per day in ETB | 101.26 | 920.47 | 2865 | 1,098.92 | 5,297.06 | 7,767.97 | 9,421.75 |
| Per day in USD | 2.44 | 22.18 | 69.04 | 26.48 | 127.64 | 187.18 | 227.03 |
| Per episode in ETB | 1,416.39 | 12,887.2 | 45,845 | 19,780.3 | 100,644 | 163,125 | 197,853 |
| Per episode in USD | 34.13 | 310.54 | 1104.7 | 476.63 | 2425.16 | 3930.73 | 4767.54 |
